# Causal Prediction of *TP53* Variant Pathogenicity Using a Perturbation‐Informed Protein Language Model

**DOI:** 10.1002/advs.202516332

**Published:** 2026-04-09

**Authors:** Huiying Chen, Yang Zhao, Boqiang Hu, Wuke Wang, Minfang Song, Annabeth Xinyu Zhao, Xiangyang Li, Gefei Wang, Yanfen Wang, Weiyan Zheng, Xinpeng Zhang, Xia Lin, Yanbin Yin, Xingxu Huang, Jinfang Zheng, Tingbo Liang

**Affiliations:** ^1^ The Key Laboratory of Pancreatic Diseases of Zhejiang Province the First Affiliated Hospital Zhejiang University School of Medicine Hangzhou China; ^2^ Research Center for Life Sciences Computing Zhejiang Lab Hangzhou Zhejiang China; ^3^ College of Science & Medicine The Australian National University Canberra Australia; ^4^ Bone Marrow Transplantation Center the First Affiliated Hospital Zhejiang University School of Medicine Hangzhou China; ^5^ Nebraska Food for Health Center Department of Food Science and Technology University of Nebraska Lincoln Nebraska USA

**Keywords:** causal, pathogenic mutations, perturbation, protein language models, TP53, variant effect prediction

## Abstract

Accurate prediction of variant functional impact is crucial for understanding human diseases, particularly for cancer‐related genes such as *TP53*. Advances in high‐throughput mutational assays have enhanced variant effect prediction (VEP), but missense classification remains challenging due to the limitations of broad, non‐gene‐specific models. Here we present CaVepP53, a *TP53*‐specific predictor fine‐tuned on perturbation‐based experimental variants. The model not only classifies mutations but also quantifies their pathogenicity by calculating Euclidean distances between the wild‐type and mutant embeddings and deriving confidence scores through logistic transformation. Benchmarking demonstrates that CaVepP53 significantly outperforms general‐purpose models, such as AlphaMissense (AM) and PrimateAI‐3D, achieving higher accuracy, precision, and F1‐score in predicting pathogenic mutations. Competitive growth assay validation of 22 mutations further confirms CaVepP53's robustness, including 7 functional novel variants absent in the ClinVar database. Thus, by integrating protein language models with experimentally validated functional data, our approach enables accurate, interpretable VEP for *TP53*, overcoming limitations of predictors trained solely on evolutionary or clinical associations. We further extended this framework to five additional cancer‐related genes (*VHL, ATM, BRCA1, RAD51C*, and *BAP1*), establishing a generalizable framework for gene‐specific VEP with potential applications in precision medicine.

## Graphical abstract

A TP53‐specific predictor, CaVepP53, is developed by fine‐tuning ESMC on experimentally validated variants, quantifying pathogenicity via Euclidean distances. It outperforms general‐purpose models and extends to five cancer genes, enabling interpretable variant classification for precision medicine.

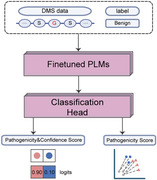

## 1 Introduction

Identifying the causal relationship between genetic variants and phenotypes is crucial for understanding the human diseases, yet remains highly challenging due to molecular complexity [1, 2]. The remarkable diversity of protein functions has led to increased research focus on missense variants that alter the amino acid sequence of proteins [3, 4, 5, 6]. However, most missense mutations remain as variants of uncertain significance (VUSs), and even when classified are often lack mechanistic insight [7, 8, 9]. High‐throughput variant determination assays, such as deep mutational scans (DMS) [10] and Perturb‐seq [11] have enabled comprehensive and reliable the effects of variants, but are difficult to scale genome‐wide [12, 13].

In parallel, protein language models (PLMs) trained on large evolutionary sequence datasets can capture the structural and functional constraints encoded in protein sequences, enabling unsupervised, zero‐shot estimation of mutation impact by comparing the probabilities of wild‐type and mutant residues [14, 15, 16]. Compared to traditional supervised approaches, which rely on labeled variant databases and are often subject to annotation biases, PLMs offer greater generalizability [17, 18]. However, general‐purpose PLMs lack gene‐level specificity and biological interpretability, limiting the models’ ability to contextualize predictions in a biologically meaningful way and limits their ability to provide mechanistically‐grounded predictions for variant effects.

Here, we report CaVepP53, a gene‐specific virtual perturbation framework for the *p53* protein, a critical tumor suppressor with well‐characterized roles in cell cycle regulation, DNA damage response, and apoptosis [19]. CaVepP53 integrates the state‐of‐the‐art (SOTA) ESMC [20] PLM with causally validated DMS data for fine‐tuning, enabling it to capture *TP53*‐specific mutational signatures. These design features allow CaVepP53 to outperform general‐purpose predictors, such as AM, [21] in benchmarking tasks focused on *TP53*. For experimental validation, we conducted a mutagenesis analysis to measure the functional impact of all possible missense mutations in *TP53*. Based on confidence scores and prior biological knowledge, 22 candidate mutations were selected for validation. Functional assays revealed that 16 of these mutations significantly altered cell growth, including 7 previously uncharacterized functional variants not listed in ClinVar. Collectively, these results demonstrate that CaVepP53 enables accurate, interpretable, and scalable variant effect prediction, offering a powerful tool for studying both known and novel mutations in *TP53*.

## 2 Results

### 2.1 CaVepP53: A *TP53*‐Specific Framework for Missense Pathogenicity Prediction and Experimental Validation

**FIGURE 1:**
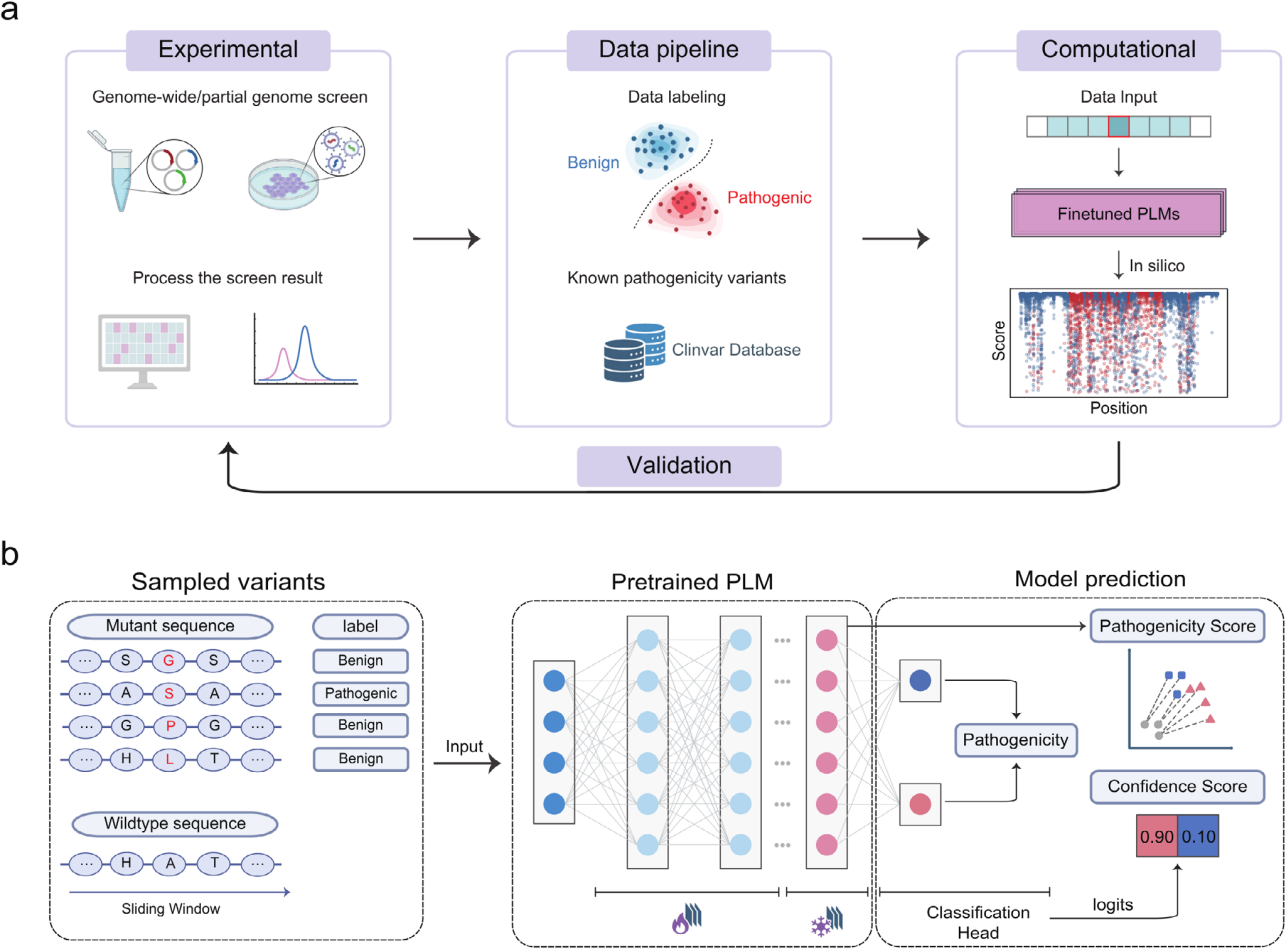
Overview of this study. (a) Workflow of CaVepP53 development and application. Experimentally validated pathogenic and benign *TP53* variants from ClinVar and DMS perturbation assays are used to fine‐tune ESMC. The trained CaVepP53 model performs saturation mutagenesis to predict pathogenicity scores for all possible *TP53* missense mutations. Candidate variants prioritized by integrated distance and confidence scores are experimentally validated via prime editing in HCT‐116 cells under Nutlin‐3a selection. (b) The *TP53*‐specific VEP model augments existing PLM with a classification head to predict the pathogenicity of variant sequences. The model primarily outputs a binary classification of variants as either benign or pathogenic, with the classification logit values serving as confidence scores. Panel **a** and b were created with BioRender.com.

To investigate the functional impact of *TP53* missense mutations through virtual perturbation, our study followed a three‐step approach: model development, pathogenicity prediction, and experimental validation (Figure 1).

#### 2.1.1 TP53‐Specific Perturbation Model Development

To realize perturbation, our study utilized pathogenic missense variants from the ClinVar [7] and DMS perturbation‐based experimental data [22] for fine‐tuning the protein language model ESMC [20]. An additional classification head was incorporated into the ESMC model to classify variants as either benign or pathogenic. The model outputs logit values that represent the predicted probabilities of pathogenicity classes, which can be interpreted as a quantitative measure of confidence in variant classification. Pathogenic score is quantitatively assessed by calculating the Euclidean distance between the embedding vectors of the wild‐type and variant sequences output by the final ESM block (Figure 1b). Additionally, the log‐odds of the predicted class in the model's output are converted into probabilities, which are then used as confidence scores (Figure 1b).

#### 2.1.2 Pathogenicity Prediction of TP53 Saturation Mutagenesis

Using the trained model, termed CaVepP53, we performed saturation mutagenesis to predict the functional impact of all possible *TP53* missense mutations. Based on pathogenicity and confidence scores, candidate variants were prioritized for downstream experiment functional validation (Figure 1a).

#### 2.1.3 Experiment Validation

Candidate variants were selected for functional experiment validation. The growth dynamics of the prime‐edited HCT‐116 cell lines harboring individual candidate variants were measured under Nutlin‐3a selection (Figure 1a).

Collectively, we present a gene‐specific variant effect prediction framework using *TP53* as a proof‐of‐concept. By integrating a protein language model with perturbation‐derived functional data, CaVepP53 enables accurate, interpretable, and scalable prediction of missense variant effects, providing a powerful tool for both basic research and precision oncology.

### 2.2 Targeted Fine‐Tuning With Functional Perturbation Data Improves Predictive Accuracy for TP53 Missense Variants

To demonstrate the advantages of gene‐specific perturbation models in predicting and characterizing pathogenic mutations, we first collected a curated perturbation dataset of functionally validated variants. Using this dataset, we then performed a focused tuning of key model parameters, including the window size, number of unfreezing layers, and PLM pretraining parameter sizes, to develop CaVepP53 with enhanced predictive capacity. We further revealed several distinct advantages of CaVepP53 over existing approaches such as AM [21] and PrimateAI‐3D [23].

#### 2.2.1 Construction of a Perturbation‐Informed Dataset Using Saturation Mutagenesis Data From DMS

Most existing VEP models rely heavily on ClinVar [7] as a source of labeled data. However, mutations in ClinVar are limited in number and primarily derived from correlation‐based clinical associations, lacking direct evidence of functional impact. Prior studies [21, 23], such as AM, have acknowledged this limitation and try to use an evolutionarily derived dataset during pretraining to correct data leakage. However, this dataset is even further removed from experimentally validated functional relevance. To improve model performance and ensure causal validity, we expanded the training dataset by integrating ClinVar with the deep mutational scanning dataset published by Stiewe et al. (hereafter referred to as the Stiewe dataset) [22]. The Stiewe dataset **c**onsists of mutations assessed through base editing technologies that quantify changes in binding affinity, thereby providing experimental evidence of functional relationships. This integration enables a shift from correlative to causally grounded variant classification, expanding the gold‐standard dataset to include 3522 single amino acid polymorphisms (SAPs), of which 1976 are labeled as pathogenic and 1546 as benign (Table S1). Considering that each residue can have up to 19 possible amino acid substitutions, we found that including all possible mutations per site was not always beneficial for model performance. In fact, in some cases, it introduced noise and reduced predictive accuracy. Empirical evaluation showed that randomly sampling 11 mutations per position yielded the best overall performance, striking a balance between data diversity and specificity (Figure S1).

#### 2.2.2 Model Optimization Through Baseline Model Selection, Fine‐Tuning Layers, and Context Size Optimization

**FIGURE 2:**
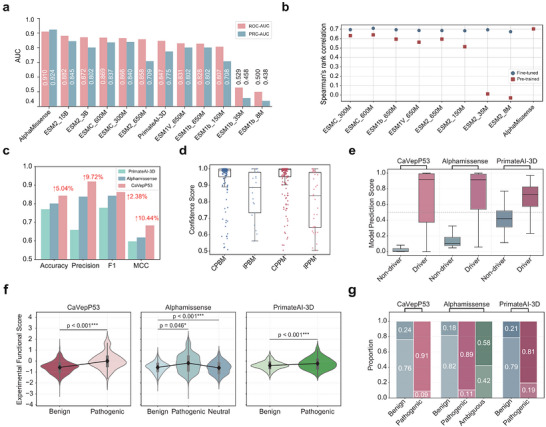
*TP53*‐specific VEP model enhances variant pathogenicity prediction accuracy. (a) Benchmarking the performance of eight protein language models based on the area under the precision‐recall curve (AUPR) using the training dataset. (b) *TP53*‐specific fine‐tuning led to substantial performance improvements across all ESM variants, with particularly rapid gains observed in smaller models such as ESM2_35 M and ESM2_8 M. (c) Performance Metrics (Accuracy, Precision, F1‐score, MCC) of AM and *TP53*‐specific model, with *TP53*‐specific model generally outperforming AM. Values above CaVepP53 bars indicate the percentage improvement over AM. (d) The model exhibits higher confidence scores for correctly predicted benign and pathogenic mutations, highlighting its strong predictive accuracy compared to inaccurate predictions. Correctly predicted benign mutations (CPBM), incorrectly predicted benign mutations (IPBM), correctly predicted pathogenic mutations (CPPM), incorrectly predicted pathogenic mutations (IPPM). (e) Distribution of prediction scores for driver and non‐driver variants across the three models (total n = 503). Box plots show median, IQR, and whiskers (1.5 × IQR). Driver vs non‐driver groups were compared using the two‐sided Mann‐Whitney U test. Sample sizes: non‐driver n = 253, driver n = 250. ^***^ *p*<0.001. (f) Distribution of experimentally measured functional scores by model‐predicted pathogenicity classes on the William independent missense set (total n = 3,945). Sample sizes for CaVepP53 binary classification, n = 3,362 (benign) and n = 687 (pathogenic); for Alphamissense, n = 475 (ambiguous), n = 2,531 (benign), n = 1,024 (pathogenic); for PrimateAI‐3D, n = 943 (pathogenic) and n = 317 (benign). Significance was assessed by two‐sided Mann‐Whitney U test (^*^ *p* < 0.05, ^**^ *p* < 0.01, ^***^ *p* < 0.001). (g) Stacked bars show the proportion of clinically annotated driver (upper) and non‐driver (lower) variants in each model's prediction category; absolute counts are displayed on the bars (total n = 503). CaVepP53 and PrimateAI‐3D use binary classification (Benign/ Pathogenic); AlphaMissense uses ternary classification (Benign/ Pathogenic/ Ambiguous).

To identify the most suitable baseline for *TP53* VEP, we first benchmarked several SOTA models on perturbation‐informed dataset. Among these, AM demonstrated the best performance (Figure 2a). However, due to the unavailability of AM's source code and the substantial computational demands of full model pretraining, we turned to the ESM family of PLMs for further development. More, ESM models offer efficient transfer learning capabilities and allow partial fine‐tuning without requiring updates to all model parameters, which drastically reduces the demand of computation. Through comprehensive test, we found that fine‐tuning only the final transformer block of ESMC was sufficient to achieve strong predictive performance (Figure S2; Figure 2b). We also observed that, in general models, performance improved with parameters size, with ESMC outperforming ESM‐2 and ESM‐1b (Figure 2b). These results are consistent with previously reported benchmarks in 3D structure prediction [20]. However, even models with relatively fewer parameters exhibited rapid performance gains when fine‐tuned on *TP53*‐specific data (Figure 2c). Among them, ESMC_600 M achieved the highest correlation with experimental measurements, matching the performance of AM. Given the importance of local sequence context in modeling, we further optimized the input window size. Five‐fold cross‐validation indicated that including 25 amino acids upstream and downstream of the mutation site yielded the best performance. In addition, fine‐tuning only the final layer yielded superior results compared with alternative parameter‐freezing strategies (Figures S3 and S4). Based on these findings, the final model, CaVepP53, was configured using ESMC_600 M, fine‐tuned on *TP53*‐specific data with a 51‐residue input window, and updating only the parameters of the last transformer block.Table 1 summarizes the hyperparameters used for model training.

### 2.3 *TP53*‐Specific Model Outperforms State‐of‐the‐Art Methods in Benchmarking and Capturing Multi‐Pathogenic Hotspot Mutations

We further evaluated the performance of CaVepP53 in distinguishing pathogenic from benign variants, comparing it with SOTA methods including AM and PrimateAI‐3D. Across several key metrics including accuracy (ACC), precision, F1‐score, and Matthews correlation coefficient (MCC), CaVepP53 consistently outperformed competing models (Figure 2c). Notably, CaVepP53 achieved a nearly 6% improvement in MCC over AM, indicating a substantial enhancement in predictive performance. The increasing performance gain may stem from two key factors: (1) the use of a *TP53*‐specific training dataset, allowing CaVepP53 to capture gene‐specific functional patterns, and (2) the integration of perturbation‐based DMS data, which introduces experimentally grounded labels with causal association. To quantitatively disentangle these two factors, we performed systematic ablation experiments using controlled training data sources and model architectures (Figure S5). Using the ESMC backbone, fine‐tuning on DMS data alone (AUROC  =  0.9396) substantially outperformed fine‐tuning on ClinVar alone (AUROC  =  0.8579), demonstrating that large‐scale, perturbation‐derived functional labels provide the dominant training signal. Combining both datasets yielded additive benefits over ClinVar alone (+0.094 AUROC). Furthermore, replacing the ESM foundation model with a conventional CNN trained on amino‐acid features led to a marked performance drop (AUROC  =  0.7426 vs. 0.9389), confirming that the pre‐trained PLM is indispensable. Collectively, these ablation studies provide direct quantitative evidence that both perturbation‐based training data and the ESM foundation model critically underpin CaVepP53's predictive accuracy. In addition, the pathogenicity scores generated by the fine‐tuned model display a more distinct bimodal distribution than those from AM, demonstrating higher specificity compared to the outcomes of the zero‐shot learning approach (Figure S6). Our model also exhibits more calibrated confidence: predictions are associated with higher confidence scores when correct and lower scores when incorrect (Figure 2d), highlighting its internal reliability.

To further evaluate the reliability of CaVepP53, we constructed a completely independent external test dataset containing 3945 missense mutations based on an additional *p53* functional dataset comprising 7467 *TP53* mutations, after rigorously excluding any variants overlapping with the internal training and test datasets. Using experimentally determined functional activity scores as the gold standard, we assessed whether variants classified as pathogenic by each model exhibited significantly lower functional scores than those classified as benign. Among the three models, CaVepP53 showed the most pronounced separation in functional score distributions between predicted pathogenic and benign groups, while all models demonstrated statistically significant differences (Figure 2f). Furthermore, analysis based on 503 clinically annotated *TP53* variants obtained from the cBioPortal database indicated (Figure 2e,g) that CaVepP53 demonstrated particularly strong consistency in identifying driver variants, achieving a ROC‐AUC of 0.918 and Cohen's d of 2.03, whereas AM and PrimateAI‐3D achieved ROC‐AUC of 0.893 and 0.865, and Cohen's **d** values of 1.94 and 1.64, respectively (Table S2).

### 2.4 Generalization to Five Additional Genes

To assess whether the CaVepP53 framework can be extended beyond *TP53*, we applied our approach to five additional genes with available perturbation data: *VHL, ATM, BRCA1, RAD51C*, and *BAP1*. For each gene, we fine‐tuned the ESMC model using a combination of ClinVar annotations and base‐editing DMS data (Table S3). To address class imbalance, we incorporated a class‐balanced cross‐entropy loss during fine‐tuning, which weights samples inversely to their class frequencies.

As shown in Figure S7, this balancing strategy consistently improved performance on imbalanced datasets such as *ATM* and *VHL*, with gains in balanced accuracy, ROC‐AUC, F1‐score, and MCC. Using the optimized training strategy, our model achieved robust performance across five three genes: ROC‐AUC values reached 0.939 for *VHL*, 0.763 for *ATM*, 0.870 for *BRCA1*, 0.968 for *RAD51C*, and 0.877 for *BAP1*, with similarly high F1‐scores and accuracies (Figure S7 and Table S3). To benchmark against existing methods, we compared our model's performance with AM and PrimateAI‐3D on the same five genes (Figure S7b–d). Our model achieved the highest ROC‐AUC on 4 out of 5 genes, with an average ROC‐AUC of 0.883 across all genes, compared to 0.841 for AM and 0.854 for PrimateAI‐3D. Similar improvements were observed in F1‐score and accuracy, where our model also outperformed both baselines on the same four genes (Figure S7c,d). The only exception was *ATM*, where AM achieved marginally higher performance. These results demonstrate that our gene‐specific perturbation framework consistently outperforms SOTA baselines across multiple genes and evaluation metrics.

Together, these results demonstrate that the gene‐specific perturbation framework developed for *TP53* transfers effectively to multiple genes, supporting its scalability and generalizability.

### 2.5 In Silico Saturation Mutagenesis Prediction of *p53*

**FIGURE 3:**
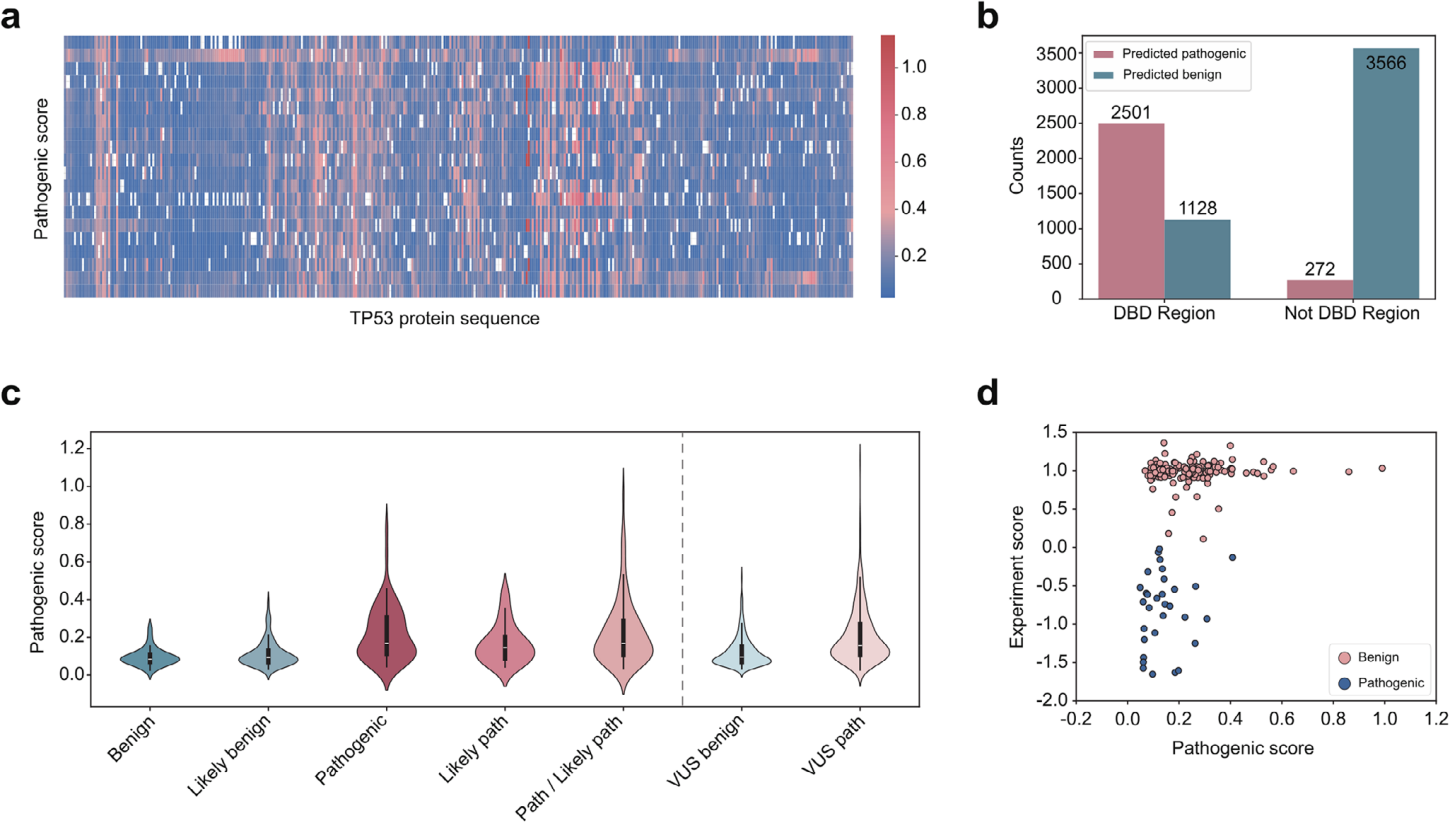
Saturation mutagenesis prediction of *p53*. (a) The heatmap for CaVepP53 pathogenicity scores of all possible amino acid substations in *TP53* protein sequence. (b) Comparative analysis of predicted pathogenic variants in DBD and Non‐DBD regions. (c) Distribution of *TP53*‐specific VEP model scores across all ClinVar labels, including classifications of VUS. (d) Prediction of experimentally validated single‐residue deletion variants. Each point represents a variant. Benign variants (n = 32) are shown in blue, and pathogenic variants (n = 125) are shown in red. Statistical significance was assessed using the two‐sided Mann–Whitney U test. ^***^ *p* < 0.001.

After confirming the high performance of our fine‐tuned *TP53*‐specific VEP model, we proceeded to conduct a saturation mutagenesis prediction analysis of the entire protein sequence (Figure 3a). Of the 7467 variant predictions generated, 2737 were classified as pathogenic and predominantly located within the DNA‐binding domain (DBD), while 4694 were benign. This distribution indicates a model bias toward identifying functionally sensitive regions such as the DBD (Figure 3a,b). To assess whether simply residing in the DBD suffices for accurate prediction, we established a biologically meaningful baseline: a rule‐based classifier that predicts pathogenicity solely based on whether a variant falls within the DBD (residues 102–292). This classifier achieved an MCC of 0.3663 and an F1‐score of 0.7623 on the same test set (Figure S8), confirming that “DBD mutation” is indeed a strong predictive feature. However, it is inherently limited: it cannot distinguish pathogenic from benign variants within the DBD, nor can it identify pathogenic mutations outside this region. In contrast, our fine‐tuned model captures these subtleties, enabling a more nuanced genome‐wide analysis. Given that the ClinVar database includes a substantial number of variants of uncertain significance (VUS), and that novel *TP53* mutations continue to be reported, we used our model to further classify these mutations. We aimed to determine whether they exhibit intermediate effects or resemble known categories but remain unclassified. Our analysis revealed that approximately 53% of ClinVar missense VUS were predicted to be benign, while 47% were predicted to be pathogenic (Figure 3c). Based on the model's strong performance with missense mutations, we extended its application to single amino acid deletions. Using a dataset of 157 experimentally validated single residue deletion variants from the Stiewe dataset, the model accurately distinguished between benign and pathogenic deletions (Figure 3d).

### 2.6 Experimental Validation of CaVepP53 Predictions on Novel and Discordant Variants

**FIGURE 4:**
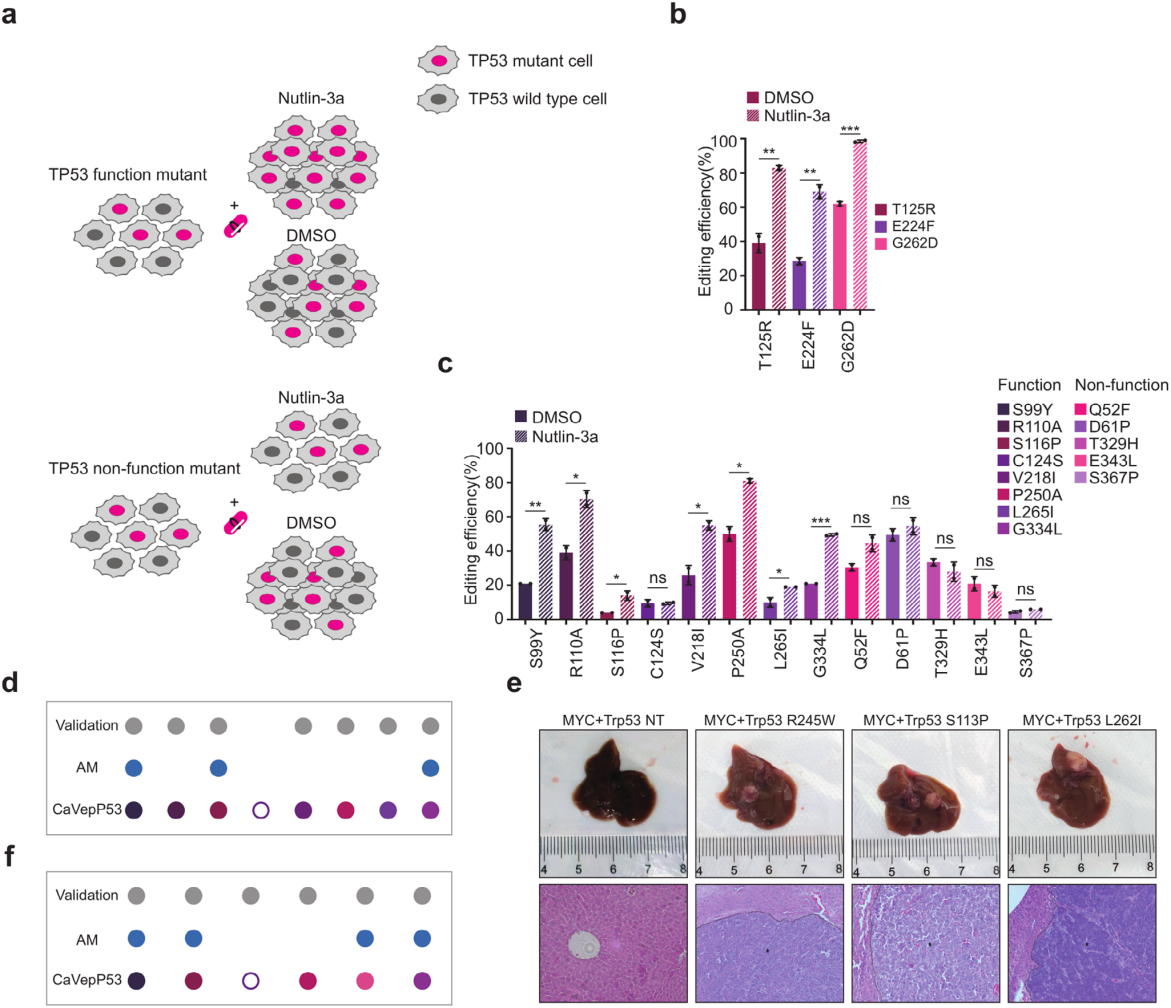
Functional validation of predicted *TP53* variants. (a) Schematic workflow: Mixed cell pools containing *TP53* prime‐edited mutants (red) and wild‐type cells (gray) were treated with DMSO or 10 µM Nutlin‐3a for 5 days. Functional mutants exhibit growth advantage under Nutlin‐3a selection, whereas nonfunctional mutants do not. (b) Editing efficiency of *TP53* mutants: 3 AM‐predicted and high‐throughput screening functional mutants. Solid bars: DMSO control; Striped bars: Nutlin‐3a treatment. (c) Editing efficiency of newly identified functional/nonfunctional mutants. Solid bars: DMSO control; Striped bars: Nutlin‐3a treatment. Shown is the mean ± s.d. abundance (n  = 2 biological replicates). A standard two‐tailed unpaired Student's t test was used for statistical analysis of the two groups. ns, not significant, ^*^ *p* < 0.05, ^**^ *p* < 0.01, ^***^ *p* < 0.001. (d) Model predictions and experimental validation of pathogenic variants: Filled circles represent variants confirmed experimentally as pathogenic. Experimental results are shown in gray, predictions by the AM model in blue, and predictions by CaVepP53 in variant‐specific colors consistent with panels b and c. Unfilled circles indicate variants where CaVepP53 predictions did not match experimental outcomes. Among 8 variants predicted pathogenic by CaVepP53, 7 were experimentally validated, whereas only 3 were predicted as pathogenic by the AM. (e) Morphology and H&E staining of a liver tumor induced by MYC; Trp53 mutation. The tumor margin is delineated by a dashed line, with the neoplastic area indicated by asterisks. (f) Reclassification of ClinVar VUS based on model prediction and experimental validation. 5 variants originally labeled as “variants of uncertain significance” (VUS) in ClinVar and Ensembl are shown. All 5 were experimentally confirmed to be functionally pathogenic. Among them, CaVepP53 correctly predicted 4, while the AM model correctly predicted 3.

Nutlin‐3a acts as an MDM2 inhibitor, stabilizing the *p53* protein and thereby promoting cell cycle arrest or apoptosis. However, *TP53* loss‐of‐function mutants, due to impaired transcriptional activity, can escape Nutlin‐3a‐induced growth suppression and continue proliferating despite drug exposure [24]. Leveraging this biological mechanism, we established a competitive growth assay to functionally validate the predictions of our fine‐tuned *TP53*‐specific variant effect predictor in prime‐edited HCT‐116 cell line. Functional mutants exhibited a significant growth advantage under drug selection, evidenced by elevated editing efficiency in co‐cultures with wild‐type cells (Figure 4a). To confirm the assay's reliability, 3 (Table S4) randomly previously characterized functional mutations, identified by AM, high throughput screening, and CaVepP53, were tested as described [22]. The results showed significantly elevated editing efficiencies following Nutlin‐3a treatment compared to controls (Figure 4b), with 100% concordance with CaVepP53 predictions. These results validated the assay's robustness in identifying functional variants, confirming the efficacy of the competition assay for distinguishing functional mutants. Following assay establishment, an additional 19 *TP53* mutants were experimentally tested (Table S4): 10 consistent with AM/screening predictions and 9 misclassified by AM (Table S5). CaVepP53 achieved 68.2% accuracy (Table S5), representing a 18.2% increase over the pretrain model (ESMC_600 M), demonstrating the efficacy of fine‐tuning. Notably, CaVepp53 demonstrated a 9.1% improvement in accuracy over the AM model on this experimental dataset. Specifically, within the subset of 9 mutations misclassified by AM, CaVeP53 correctly predicted 6, achieving an accuracy of 66.7% (6/9) on these challenging cases (Table S5). Critically, we identified 7 novel functional variants (S99Y, R110A, S116P, V218I, P250A, L265I, G334L) and 5 novel nonfunctional (Q52F, D61P, T329H, E343L, S367P) mutants (Figure 4c), including 3 VUS from ClinVar and Ensembl (S99Y, S116P, P250A). Notably, the CaVepP53 model demonstrates exceptional capability in identifying pathogenic mutations (Figure 4d). Besides, three mutants (S116P, P250A, and L265I) were randomly selected for assessment of liver tumorigenesis. Furthermore, three mutants (S116P, P250A, and L265I) were randomly selected to evaluate their potential for liver tumorigenesis in mice. Both the S116P (corresponding to mouse S113P) and L265I (mouse L262I) mutants induced malignant transformation in MYC‐expressing hepatocytes, consistent with the established oncogenic mutant R248W (mouse R245W), thereby confirming their tumorigenic potential in vivo (Figure 4e). These results provide causal evidence for VUS reclassification and advance *TP53* functional annotation (Figure 4f). In summary, CaVepP53 demonstrated enhanced predictive performance, accurately classifying 15 out of 22 validated variants, including resolving VUS cases such as S116P and P250A, thereby supporting evidence‐driven reannotation.

## 3 Discussion

In this study, we developed a *TP53*‐specific variant effect predictor, CaVepP53, by fine‐tuning ESMC‐600 M on DMS and ClinVar data, achieving high performance in classifying pathogenic and benign variants. CaVepP53 outperformed existing tools like AM and PrimateAI‐3D, and correctly predicted 22 mutations, which were supported by cell growth experimental validation, including 7 novel functional mutations. To enhance interpretability, we introduced an autonomous AI agent that simulates human‐like reasoning by integrating literature, structural data, and domain‐specific databases. This framework enables scalable functional interpretation and experimental design. Our study presents the first gene‐specific perturbation variant prediction model for *TP53*, supported by an LLM‐based agent system, with potential for expansion to broader perturbation types, gene networks, and disease contexts.

Our study introduces two key innovations in VEP for *TP53*.

1. Trade‐off between gene‐specific and general models. By tailoring our model specifically to *TP53*, we achieved consistently superior performance compared to existing general models such as AM [21] and ESMv [16]. This improvement likely stems from the fact that, while genes may share common mutational patterns, each gene also possesses unique features that general models may overlook. A gene‐specific approach enables the model to capture these distinctive characteristics, offering a framework that can be extended to other genes of interest.
2. Leveraging perturbation‐derived data for mechanism‐informed VEP. We leverage large‐scale perturbation datasets derived from CRISPR‐based DMS, rather than relying solely on evolutionary conservation or clinical annotations, as is common in many existing VEP models such as AM and ESM‐based predictors. Those approaches often depend on indirect signals, including assumptions that rare variants are more likely to be pathogenic, which may introduce bias and do not directly measure functional impact. In contrast, our DMS‐derived training data are linked to measurable phenotypes, such as changes in protein binding affinity, enabling the model to learn causal associations and substantially improve predictive accuracy. These perturbation‐derived data directly quantify the functional consequences of individual variants and therefore approximate intervention outcomes rather than correlations. These features position CaVepP53 as a mechanism‐informed predictor: its value lies not only on predictive performance, but also on its ability to link sequence variation to experimentally grounded functional effects in a manner that is conceptually distinct from conventional fine‐tuning paradigms.

This study also has two limitations. (1) The dynamic nature of cellular systems is not explicitly captured. Although the gene‐specific training data partially reflect biological context, they do not account for condition‐specific pathway or cell state variability. (2) The model is currently unable to assess the functional consequences of synonymous mutations, which may still affect gene expression, splicing, or translational efficiency.

Lastly, several directions can be considered to further improve model performance and biological relevance. (1) Integrating cell type specific dynamic regulatory networks could enhance the model's ability to capture context dependent mutational effects. (2) Incorporating training data linked to more direct phenotypic outcomes, such as changes in cell proliferation, apoptosis, or in vivo tumorigenicity, may strengthen causal associations and reduce false positives. (3) Extending the framework to incorporate genome‐scale models could enable the prediction of functional consequences for synonymous mutations, which are currently not captured but may exert regulatory effects.

## 4 Experimental and Methods Section

### 4.1 Dataset Preparation

The fine‐tuning of the ESMC pathogenicity classification model was performed using variant annotation datasets from publicly available experimental data and the ClinVar database. The experimental data was derived from a study on saturated mutagenesis of the primary *TP53* DBD [22]. The curated dataset has been designated as the Stiewe dataset. The experimental data were labeled for pathogenicity based on the Relative Functional Score (RFS) provided in the study, with variants classified as pathogenic if the RFS > 0 and benign if the RFS ≤ 0. For the ClinVar dataset, variants annotated as ‘Benign’, ‘Likely benign’, or ‘Benign/Likely benign’ were categorized as benign, while those labeled as ‘Pathogenic’, ‘Likely pathogenic’, or ‘Pathogenic/Likely pathogenic’ were classified as pathogenic. The Stiewe dataset contributed 3419 single amino acid polymorphisms (SAPs), of which 1955 were classified as pathogenic and 1464 as benign. While the ClinVar set provided 327 SAPs, with 203 identified as pathogenic and 124 as benign. This approach ensures that the model is fine‐tuned using well‐established, high‐quality data, enabling it to make reliable predictions on novel variants. Variant data from these datasets were merged, with duplicates removed and conflicting data filtered out to ensure data integrity and consistency. Together, these datasets encompassed a total of 3522 SAPs, with 1976 labeled as pathogenic and 1546 as benign. In our study, variant sequences were analyzed using a window size of 51 amino acids (AA), with 25 AA extended upstream and downstream of the mutation site. If the upstream or downstream sequence length was less than 25 AA, a special padding token (<pad>) was used to ensure consistent sequence length. During the training process, the 3522 annotated data entries were randomly partitioned into five equal subsets. Each subset was used once as the validation set, while the remaining four subsets served as the training set. The model that demonstrated the best performance on its respective validation set was selected for comparison with the AM model in the final evaluation phase. The specific validation set used during this model's training was retained for the final comparison, ensuring that the evaluation was conducted under conditions reflective of the model's optimization phase. This approach ensured that the model selection was based on robust cross‐validation, thereby enhancing the reliability of the comparative analysis.

To enhance the generalizability of our model, we incorporated experimental deep mutational scanning (DMS) data for five additional genes: *VHL* [25], *ATM* [26], *BRCA1* [12], *RAD51C* [27], and *BAP1* [28]. These datasets were obtained from previously published saturation mutagenesis studies. For each gene, variant pathogenicity labels were assigned according to the thresholds defined in the original studies to ensure consistency with the experimental context of each assay.

### 4.2 Dense Layer‐Based Mutation Classification

In the model training process, pathogenic variant sequences were labeled as 1, while benign variant sequences were labeled as 0. These labeled data were subsequently used to train the classification model. To fine‐tune the pre‐trained PLM, ESMC, [20] a fully connected layer was added to the model, which was responsible for outputting predictions regarding the pathogenicity of each variant. The fine‐tuning process aimed to optimize the model's parameters, enabling it to accurately classify variants based on the labeled pathogenicity data.

For fine‐tuning, we performed experiments with various layers of the model for unfreezing, and ultimately, we focused on fine‐tuning the final layer of the ESM block as well as the classification head. Specifically, the fine‐tuning was conducted by minimizing the cross‐entropy loss function, which is commonly used for classification tasks. The cross‐entropy loss, denoted as *L_CE_*, is defined as:

$$L_{CE}=-\frac{1}{N}\sum_{i=1}^{n}\left[y_{i}log\left({\hat{y}}_{i}\right)+\left(1-y_{i}\right)log\left(1-{\hat{y}}_{i}\right)\right]$$

where *N* represents the total number of samples, *y_i_* is the true label (1 for pathogenic and 0 for benign), and $\hat{y_{i}}$ is the predicted probability for the corresponding sample.

To address the inherent class imbalance between pathogenic and benign variants, we employed a weighted binary cross‐entropy loss function during training. The weighted loss function is defined as:

$$L_{WCE}=-\frac{1}{N}\sum_{i=1}^{n}\left[\omega_{1}y_{i}log\left({\hat{y}}_{i}\right)+\omega_{0}\left(1-y_{i}\right)log\left(1-{\hat{y}}_{i}\right)\right]$$

where ω_0_ and ω_1_ represent weight coefficients for negative (benign) and positive (pathogenic) classes, respectively. These weights were computed using a balanced weighting strategy, where each weight is inversely proportional to the frequency of its corresponding class: ω_*c*_ = *N*/(2·*N_C_*), with *N* denoting the total number of training samples and *N_C_* indicating the sample count for class *c*. This weighting scheme mitigates bias toward the majority class, thereby enhancing sensitivity in detecting pathogenic variants. By minimizing this loss function, the model adjusts its weights to achieve optimal classification accuracy for predicting the pathogenicity of *TP53* variants.

To optimize model performance and avoid overfitting, we employed k‐fold cross‐validation (k = 5) during the training process. Specifically, the dataset was randomly split into 5 subsets. For each iteration, one subset was used as the validation set, and the remaining 4 subsets were used for training. This cross‐validation approach, repeated across multiple iterations, provides an estimate of the model's generalization ability and ensures consistent performance across different data partitions.

Model tuning was conducted based on performance metrics such as the F1 score, Area Under the Receiver Operating Characteristic Curve (AUC‐ROC), and Precision. The F1 score is a balanced metric that combines both precision and recall, particularly useful when dealing with imbalanced datasets. The F1 score is calculated as the harmonic mean of precision and recall:

$F1\;\;\mathrm{Score}=2\times \frac{\mathrm{Precision}\times \mathrm{Recall}}{\mathrm{Precision}+\mathrm{Recall}}$ where precision (the positive predictive value) and Recall (sensitivity) are defined as:

$\mathrm{Precision}=2\times \frac{\mathrm{TP}}{\mathrm{TP}+\mathrm{FP}},\;\mathrm{Recall}=2\times \frac{\mathrm{TP}}{\mathrm{TP}+\mathrm{FN}}$ where TP, FP, and FN represent true positives, false positives, and false negatives, respectively.

The model's overall discriminative ability was summarized by the AUC‐ROC. The ROC curve plots the true positive rate (Recall) against the false positive rate (1‐Specificity) across all decision thresholds. The AUC‐ROC value represents the probability that the model ranks a randomly chosen positive instance higher than a randomly chosen negative one, with a higher value indicating better classification performance.

Using cross‐validation, hyperparameters were selected to optimize the balance among these metrics (Precision, Recall, F1 score, and AUC‐ROC), ensuring a robust and accurate model.

### 4.3 Model Training

The model was fine‐tuned using the AdamW optimizer with an initial learning rate of 4e‐5 and a weight decay of 1e‐5 to prevent overfitting. We employed a linear decay learning rate scheduler with a warmup period of 10% of the total training steps, allowing the model to gradually adapt to the target task.

The model was trained for a maximum of 3 epochs with early stopping triggered if the validation loss did not improve by at least 0.0001 for 5 consecutive evaluation steps. Training utilized automatic mixed precision (FP16) to accelerate computation while maintaining numerical stability.

Model performance was rigorously evaluated through 5‐fold cross‐validation with a fixed random seed (42) to ensure reproducibility. In each fold, the base ESMC model was fine‐tuned on the training split, while hyperparameter tuning and model selection were performed on the validation split. Following cross‐validation, the optimal number of training steps was determined by averaging the best‐performing steps across all folds. This optimal step count was then used to train the final model on the entire training dataset (Table 1).

**TABLE 1:** Hyperparameter settings for ESMC‐based variant classification model.

Hyperparameter	Value / Setting	Type / Description
**Learning rate**	4e‐5	Continual (AdamW optimizer)
**Weight decay**	1e‐5	L2 regularization
**Dropout rate**	0.1 (classifier head)	Regularization
**Batch size**	16	Discrete
**Gradient accumulation steps**	2	Effective batch size = 32
**Training epochs**	3 (maximum)	Early stopping applied
**Early stopping patience**	5 evaluation steps	Stop if no improvement
**Early stopping threshold**	0.0001	Minimum improvement
**Warmup steps**	10% of total steps	Learning rate scheduling
**Learning rate scheduler**	Linear decay with warmup	Optimization
**Cross‐validation folds**	5	Performance evaluation
**Random seed**	42	Reproducibility
**Loss function**	Weighted cross‐entropy	Classification

### 4.4 Model Output and Pathogenicity Prediction

After fine‐tuning, the ESMC model outputs a confidence score that represents the predicted pathogenicity of each input mutation sequence. This confidence score is derived from the model's logit values, which correspond to the logits of the mutation being classified into pathogenic (1) or benign (0) categories. The logit score is a raw output from the model's classification layer and represents the unscaled log‐odds of the mutation being pathogenic.

The model outputs a logit for each input sequence, which is transformed via a sigmoid function to yield a pathogenicity probability **P ∈ (0,1)**. Values near 1 indicate high confidence in pathogenicity, while values near 0 indicate benignity. In this study, we use 0.5 as the classification threshold.

This confidence score offers an interpretable measure that reflects the model's certainty about the mutation's functional impact. Higher logit values (resulting in higher confidence scores) indicate stronger evidence supporting the pathogenicity of the mutation, while lower logit values indicate weaker evidence. This probabilistic output thus provides a nuanced and quantifiable estimate of the mutation's pathogenic potential, supporting more informed decision‐making in genetic variant interpretation.

To enhance the interpretability of the model's predictions, we further introduced Euclidean distance as a measure of similarity between the embeddings of wild‐type and variant sequences. Specifically, for each mutation, the model calculates the average embedding of both the wild‐type and mutant sequences, using a sliding window of 51 AA around the mutation site. This ensures that both the mutation site and its local sequence context are considered when estimating the effect of the mutation.

The average embedding for a sequence is calculated as the mean vector of all token embeddings within the window, which can be expressed mathematically as:

$$E_{avg}=\frac{1}{N}\sum_{i=1}^{N}e_{i}$$

where *e_i_* represents the embedding of the *i*‐th amino acid within the 51 AA window, and N is the total number of amino acids within the window (in this case, N = 51).

Once the average embeddings for both the wild‐type and variant sequences are obtained, we compute the Euclidean distance between them to quantify the dissimilarity between the two sequences. The Euclidean distance *d* is given by the following formula:

$$d=\sqrt{\sum_{i=1}^{N}{\left(x_{i}-y_{i}\right)}^{2}}$$

where *x_i_* an *y_i_* represent the embedding values of the wild‐type and variant sequences, respectively, at each dimension of the embedding space. A larger Euclidean distance indicates a greater divergence between the wild‐type and mutant sequences, suggesting a higher likelihood that the mutation will disrupt protein function. In this context, a large distance is often associated with pathogenic mutations, as it suggests that the mutation induces a significant structural or functional change in the protein.

To explore the basis of model predictions, we examined the Euclidean distance between wild‐type and mutant embeddings. While this distance provides a continuous measure of representation shift, we observed that it correlated only modestly with experimental functional scores in our validation sets. This suggests that while the distance metric offers interpretability by quantifying sequence‐level perturbation, additional factors, such as local structural context, may be needed to fully capture functional impact.

### 4.5 Learnable Embedding CNN Baseline

To establish a non‐pretrained baseline, we implemented a lightweight CNN with a learnable embedding layer (LearnableEmb‐CNN). Each protein sequence was tokenized into integers (1–20, with 0 for padding) and passed through a randomly initialized embedding layer (vocab size 21, dimension 128) that is jointly optimized during training, allowing task‐specific amino acid representations to emerge without relying on pre‐trained models or biophysical features. Learnable positional encodings were added before three 1D convolutional blocks (kernel sizes 3, 5, 7; channels 128→ 64→ 128→ 256), each followed by batch normalization, ReLU, and dropout (p = 0.3). Global max pooling was applied, and two fully connected layers (256→ 128→ 2) produced binary classification logits. The model was trained with cross‐entropy loss using balanced class weights, Adam optimizer (lr = 1 × 10^−^^4^), batch size 32, and early stopping (patience = 5) based on validation F1, under 5‐fold cross‐validation. This baseline (∼1 M parameters) quantifies task‐specific learning without external knowledge, enabling controlled comparison with pLM‐based models in our ablation study.

### 4.6 Selection of Mutation for Validation

Our variant selection approach is designed to identify potential de novo mutations. After validating the reliability of our experimental results with known positive and negative control variants, candidate variants were selected according to predefined criteria rather than random sampling: 1) are predicted to be pathogenic by our model but are labeled as VUS in ClinVar/COSMIC/Ensembl/NCI databases; 2) are not covered by experimental data; and 3) show discordant predictions between the AM and ours. This strategy ensures the comprehensive evaluation of our model's predictive accuracy and provides a basis for discovering novel potentially pathogenic mutations.

### 4.7 Plasmid Construction

The pegRNA and nick‐sgRNA plasmids are constructed according to the methods described in our previous study [29]. The detailed methods are specified as follows. The pegRNA plasmid backbone is amplified from pGL3‐U6‐sgRNA‐EGFP (Addgene, #107721) and then cut by BsaI‐HFv2 (NEB) for overhangs. Spacer oligos, pegRNA 3’ extension oligos, and sgRNA scaffold oligos are synthesized with compatible overhangs. Next, the four fragments are assembled with T4 DNA Ligase (NEB). General primers for constructions and sequences of pegRNAs are listed in Data S1 and S2. PE7 plasmid was purchased from Addgene (Addgene, #214812).

### 4.8 Generation of *TP53* Mutant Cells

HCT‐116 cells were seeded in 24‐well plates at 50% confluency. After 12 h of culture, a transfection mixture containing 0.9 µg PE7 plasmids, 0.3 µg pegRNA‐EGFP plasmids, 0.1 µg nick‐sgRNA‐mCherry plasmids, and 3 µl transfection reagent was prepared and added to the plates. Cells were incubated with the mixture for 24 h. Two days post‐transfection, EGFP/mCherry double‐positive cells were isolated by flow cytometry for subsequent experiments.

### 4.9 Competition Assays

Nutlin‐3a was dissolved in dimethyl sulfoxide (DMSO) to prepare a 40 mm stock solution. *TP53*‐mutant HCT‐116 cells (4000 cells/well in 24‐well plates) were treated with 10 µm Nutlin‐3a or vehicle control (DMSO) for 5 days. This assay exploits the established mechanism whereby functional *TP53* mutants evade Nutlin‐3a‐induced cell cycle arrest and apoptosis due to impaired transcriptional activity, whereas nonfunctional mutants succumb to these effects. The functional *TP53* mutants exhibited a growth advantage and showed increased editing efficiency upon Nutlin‐3a treatment. Cells were then harvested, and *TP53* alleles were PCR‐amplified and Sanger‐sequenced. Primers used are listed in Data S3. The mutant allele frequency was calculated by EditR [30] and compared between Nutlin‐3a‐treated and DMSO control groups.

### 4.10 Generation of Mouse Liver Tumors

Hydrodynamic injection was performed using a solution prepared in 10% (v/w) normal saline, containing a mixture of 5 µg MYC‐luciferase plasmid, 1 µg SB100X plasmid, 30 µg PE7 plasmid, 15 µg pegRNA‐EGFP plasmid, and 15 µg nick‐sgRNA‐mCherry plasmid. Mice were euthanized upon tumor development, as confirmed by luciferase bioluminescence imaging.

### 4.11 HE Staining

Paraffin‐embedded sections were dewaxed in xylene and rehydrated through a graded ethanol series, followed by hydration in distilled water for ten minutes. After dewaxing, the sections were stained with hematoxylin until nuclei were clearly visualized, rinsed twice with distilled water, and then treated with ammonia water for bluing. Subsequently, the sections were counterstained with eosin, dehydrated through a graded ethanol series, cleared in xylene, and finally mounted.

### 4.12 Materials Table

**Table:** 

REAGENTS	SOURCE	IDENTIFIER
**Chemicals**		
Nutlin‐3a	MCE	HY‐10029
Dimethyl sulfoxide	MCE	HY‐Y0320C
EZ Trans	Life‐iLab	AC04L092
QuickExtract DNA Extraction Solution	LGC(Lucigen)	QE09050
Phanta Max Super‐Fidelity DNA Polymerase	Vazyme	P505‐d1
**Cell Lines**		
HCT‐116	ZQXZBIO	ZQ0125

### 4.13 Statistical Analyses

Python (v3.13) and GraphPad Prism was used for graphical representation and statistical analysis of data. Data are presented as mean ± SD from two independent biologic replicates (n = 2 per group). For comparisons between ewo groups, a standard two‐tailed unpaired Student's t test was applied under the assumption of normal distribution and equal variances. For non‐normally distributed data, the Mann‐Whitney U test was used. Significans levels are indicated as ns (not significant, p**≥**0.05), ^*^ *p*<0.05, ^**^ *p*<0.01, ^***^ *p*<0.001.

## Author Contributions

T.B.L., J.F.Z., and X.X.H. conceived and supervised the project. H.Y.C. and B.Q.H. built the CaVepP53 framework. H.Y.C. built and trained the AI models with the assistance of W.K.W., Q.Y.Z., and M.F.S. Y.Z. and H.Y.C. designed and performed biochemical experiments with the assistance of X.Y.L., G.F.W., Y.F.W., W.Y.Z., and X.L. H.Y.C., Y.Z., H.P.Z., Y.B.Y., J.F.Z., and X.X.H. analysed the data. H.Y.C, Y.Z., J.F.Z., X.X.H. and T.B.L. wrote and revised the manuscript.

## Funding

National Natural Science Foundation of China (Grant No. 82450113), the National Key R&D Program of China (2024YFA1306400), “Pioneer” and “Leading Goose” R&D Program of Zhejiang (2024SSYS0007), the Key Research Program of Chinese Academy of Sciences (ZDBS‐ZRKJZ‐TLC008), and the Jiangsu Basic Research Center for Synthetic Biology Grant (BK20233003).

## Ethics Statement

All animal experiments were approved by the Laboratory Animal Center Committee of the First Affiliated Hospita, Zhejiang University School of Medicine (Laboratory Animal Use License No.2023‐0009) and were conducted in accordance with the Guide for the Care and Use of Laboratory Animals.

## Conflicts of Interest

The authors declare no conflicts of interest.

## Supporting information




**Supporting File**: advs75089‐sup‐0001‐SuppMat.docx.

## Data Availability

The data that support the findings of this study are openly available in github at https://github.com/HuangLab‐Bioinformatics‐zju/CaVepP53, reference number 0.
