# Supplementary material for: Causal Prediction of TP53 Variant Pathogenicity Using a Perturbation‐Informed Protein Language Model
Source: Adv Sci (Weinh). 2026 Apr 9;13(34):e16332. doi: 10.1002/advs.202516332 (PMC13285122; doi:10.1002/advs.202516332)
Supplement: Supplementary file 1 — Supporting File: advs75089‐sup‐0001‐SuppMat.docx. [file ADVS-13-e16332-s001.docx]

Supplementary Information for

**Causal Prediction of *TP53* Variant Pathogenicity Using a Perturbation-informed Protein Language Model**

Huiying Chen^1,†^, Yang Zhao^1,†^, Boqiang Hu^1,†^, Wuke Wang^1^, Minfang Song^2^, Annabeth Xinyu Zhao^3^, Xiangyang Li^1^, Gefei Wang^1^, Yanfen Wang^1^, Weiyan Zheng^4^, Xinpeng Zhang^5^, Xia Lin^1^, Yanbin Yin^5^, Xingxu Huang^1,^*, Jinfang Zheng^2,^*, Tingbo Liang^1,^*

Corresponding author:

Xingxu Huang, The Key Laboratory of Pancreatic Diseases of Zhejiang Province, the First Affiliated Hospital, Zhejiang University School of Medicine, Hangzhou 310003, China. E-mail: [huangxx@shanghaitech.edu.cn](mailto:huangxx@shanghaitech.edu.cn)

Jinfang Zheng, Research Center for Life Sciences computing, Zhejiang Lab, Hangzhou 311121, Zhejiang, China. E-mail: [zhengjinfang1220@gmail.com](mailto:zhengjinfang1220@gmail.com)

Tingbo Liang, The Key Laboratory of Pancreatic Diseases of Zhejiang Province, the First Affiliated Hospital, Zhejiang University School of Medicine, Hangzhou 310003, China. E-mail: [liangtingbo@zju.edu.cn](mailto:liangtingbo@zju.edu.cn)

**The file includes:**

Supplementary Text

Figs. S1 to S8

Tables S1 to S5

**
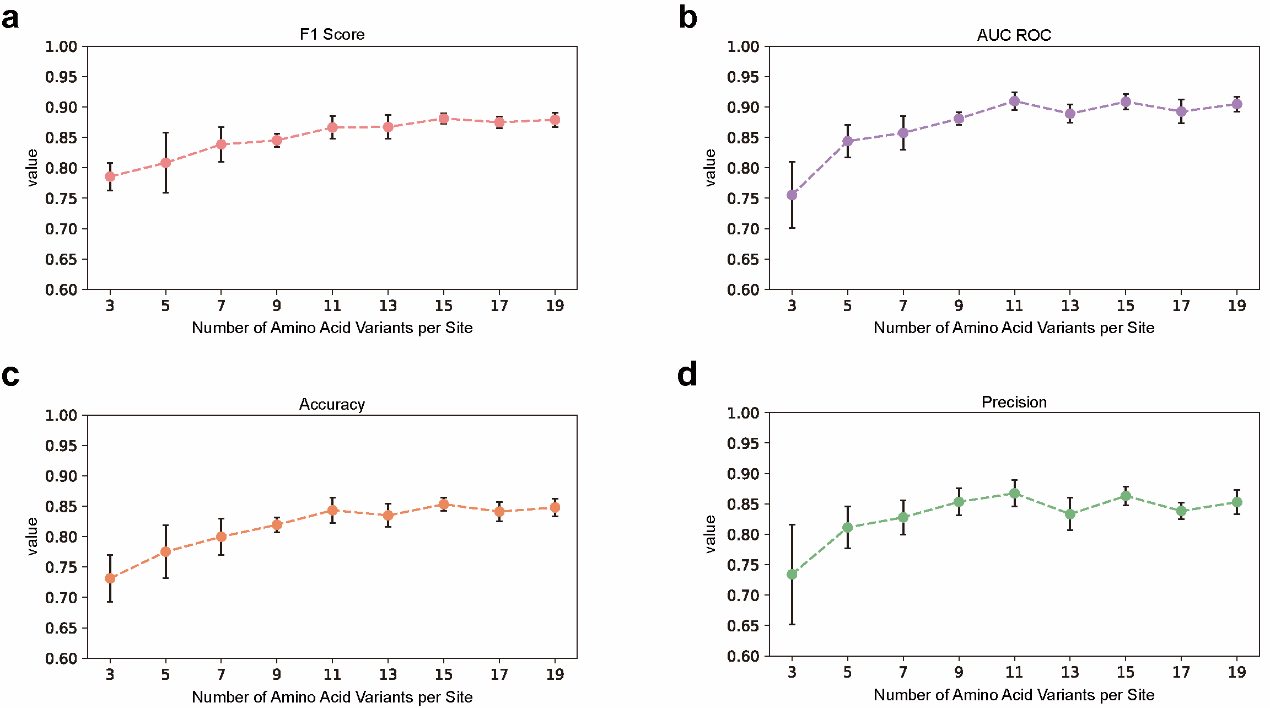
**

**Fig. S1 | Impact of amino acid variant data volume on model performance. a**, F1 Score, **b**, AUC-ROC, **c**, Accuracy, and **d**, Precision are plotted against the number of amino acid variants per site, illustrating the model's performance metrics. The x-axis represents the number of randomly selected amino acid variant annotations per site, ranging from 3 to 19. Data are presented as mean ± 95% CI from five-fold cross-validation. As the data volume increases, there is a notable improvement in all performance metrics. However, beyond half the number of possible variants per site (9-12), the enhancement in performance becomes marginal, indicating diminishing returns on additional data. Error bars denote the confidence intervals, reflecting the variability of the estimates.

**
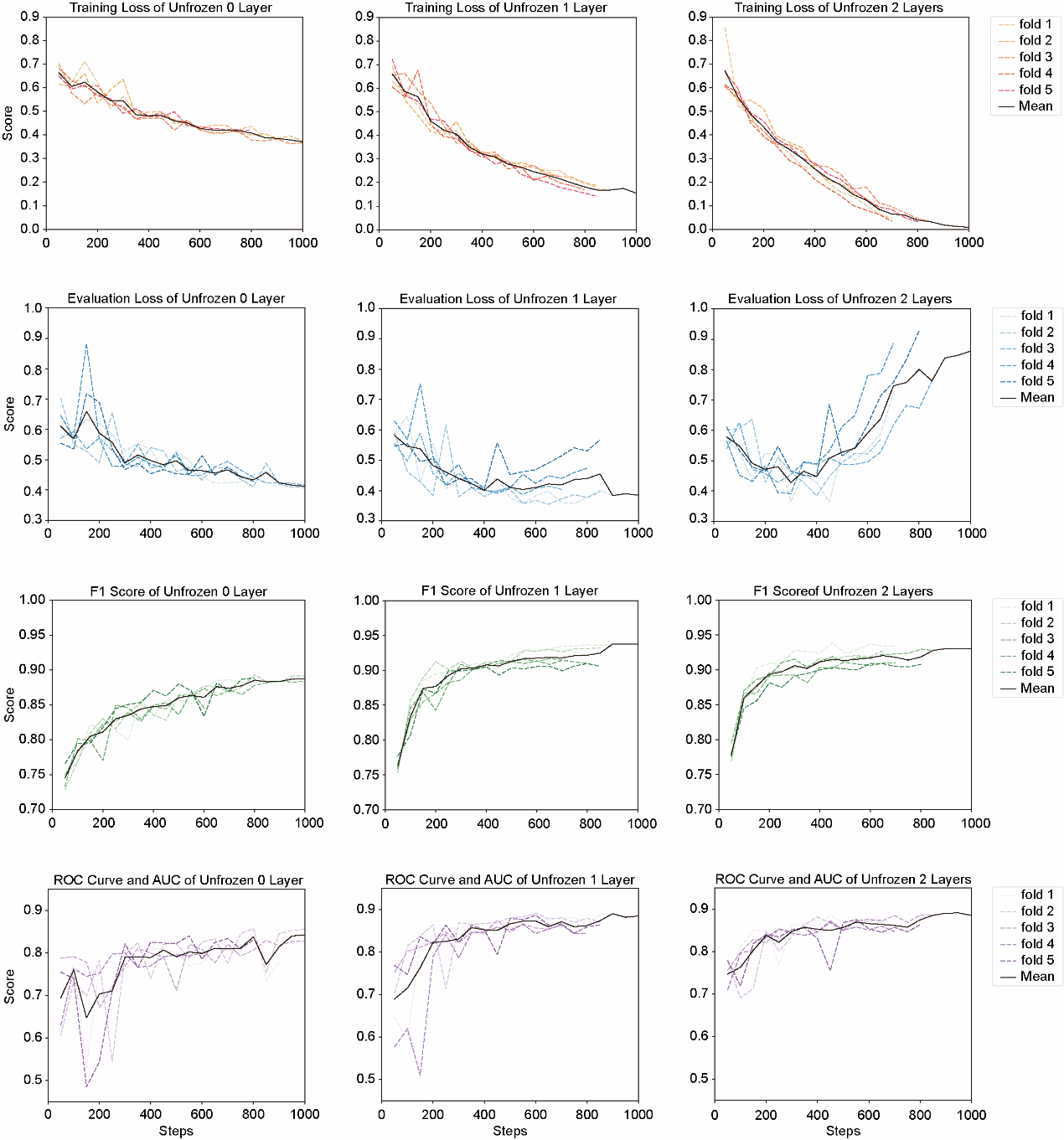
**

**Fig. S2 | Performance comparison of models with different unfreezing layers.** Performance trends of models under varying numbers of unfrozen layers (0, 1, 2) during training and evaluation. The metrics include training loss, evaluation loss, F1 score, ROC curve, and AUC value. Solid lines represent the mean performance, while dashed lines depict individual fold performance. Models with unfrozen ESM blocks exhibit better fitting but overfitting.

**
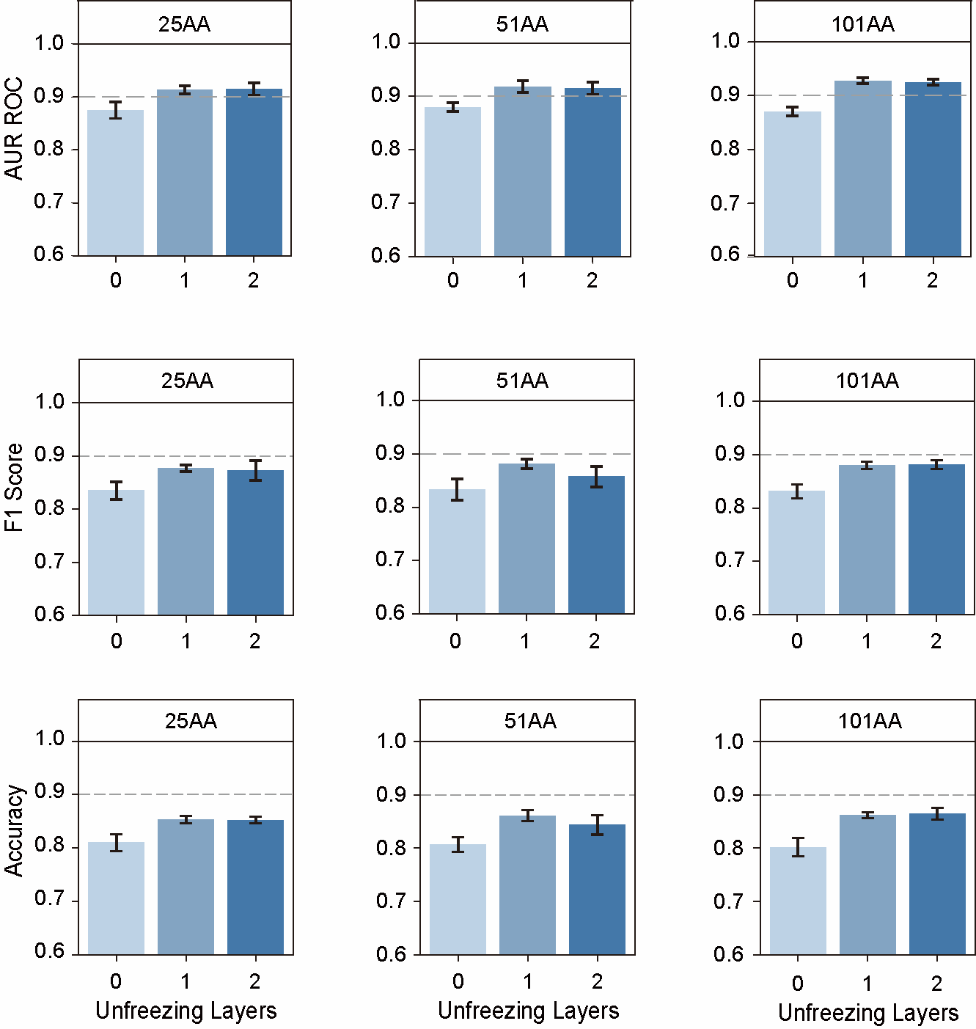
**

**Fig. S3 | Performance evaluation of sequence-based pathogenicity classification across five-fold cross-validation.** The bar chart illustrates the average performance of AUC-ROC, F1 score, and accuracy during five-fold cross-validation. Each subplot represents the impact of different unfreezing layer counts (0, 1, 2) on model performance, with error bars indicating the standard error. An unfreezing layer count of 0 indicates fine-tuning only the newly added classification layer.

**
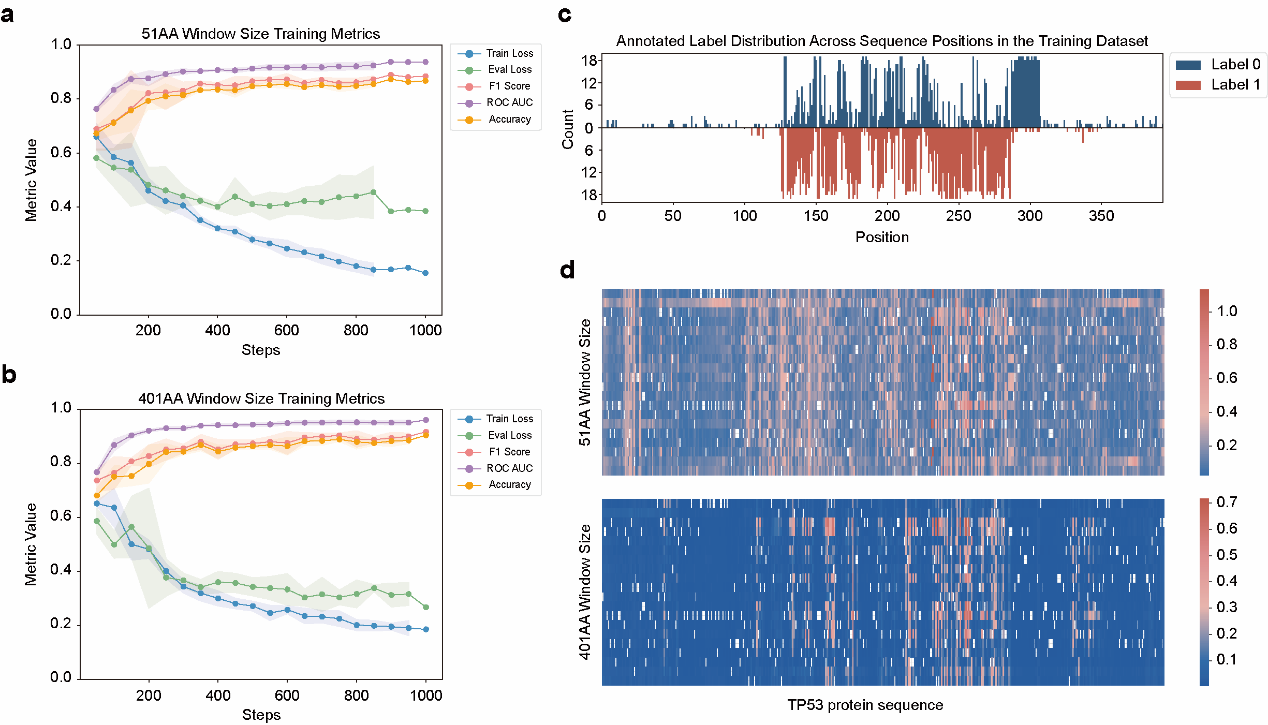
**

**Fig. S4 | Comparative analysis of TP53 mutation effects across different context window sizes.** **a**, Training metrics for the 51AA window size, illustrating the performance over training steps. **b**, Training metrics for the 401AA window size, showing a slightly better performance compared to the 51AA size. **c**, Distribution of annotated labels across sequence positions in the training dataset, with Label 0 representing benign mutations and Label 1 indicating pathogenic mutations. **d**, Distribution of pathogenic scores post-training for the 51AA and 401AA window sizes. The 401AA window size shows a tendency to assign benign scores due to overfitting, particularly evident in regions with less data distribution as seen in panel **c**.


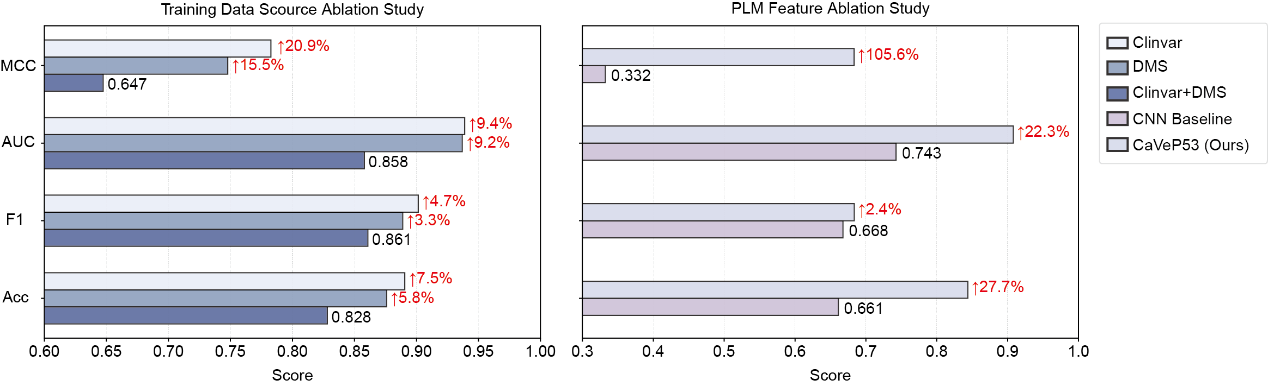


**Fig. S5** | **Ablation studies on training data sources and protein language model features for CaVeP53 performance. a, Training data source ablation.** Models were trained on ClinVar only (n=110), DMS only (n=3,202), or combined ClinVar+DMS data, and evaluated on the ClinVar test set. Percentages indicate gains of combined training relative to single-source training. **b, PLM feature ablation.** Comparison of CNN baseline (traditional amino acid features) versus ESMC-based models with or without PLM pre-training. Percentages indicate improvements of CaVeP53 relative to CNN baseline.


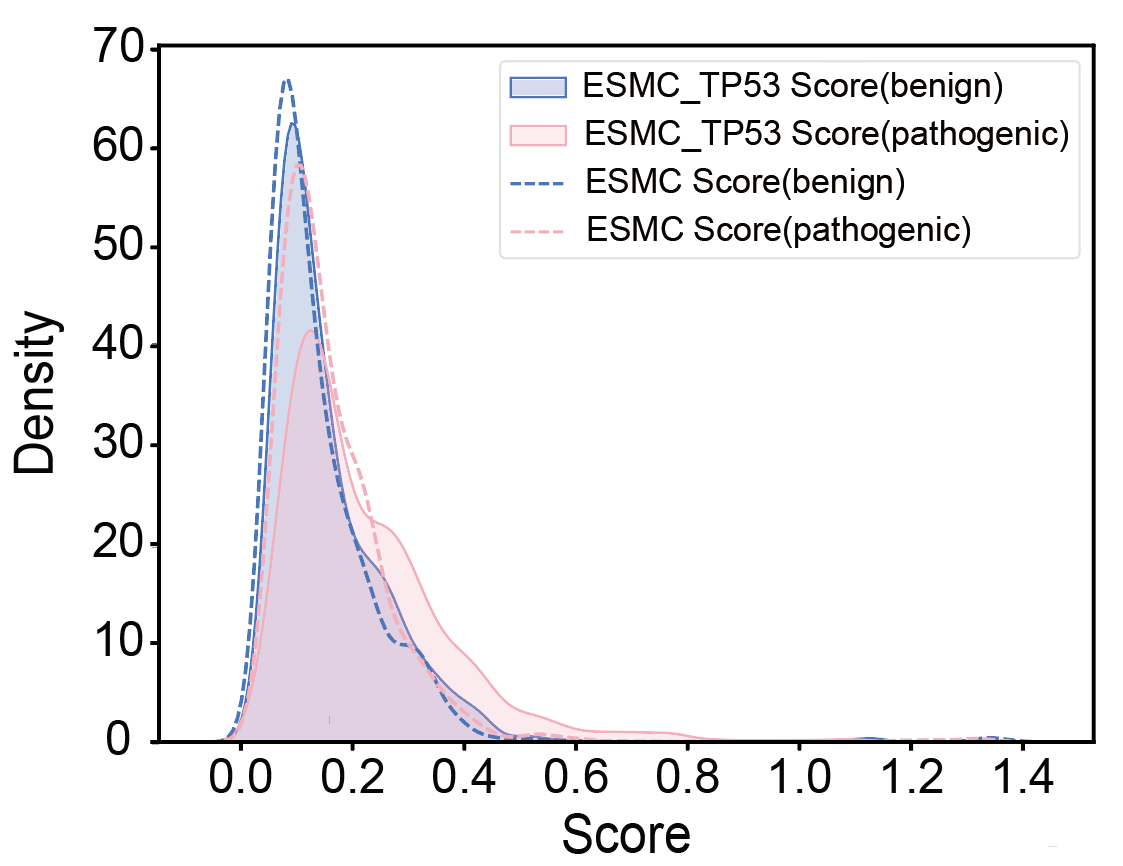


**Fig. S6 | Distribution of pathogenic score**. A comparison of pathogenic score distributions for benign and pathogenic variants under TP53-specific and base models.


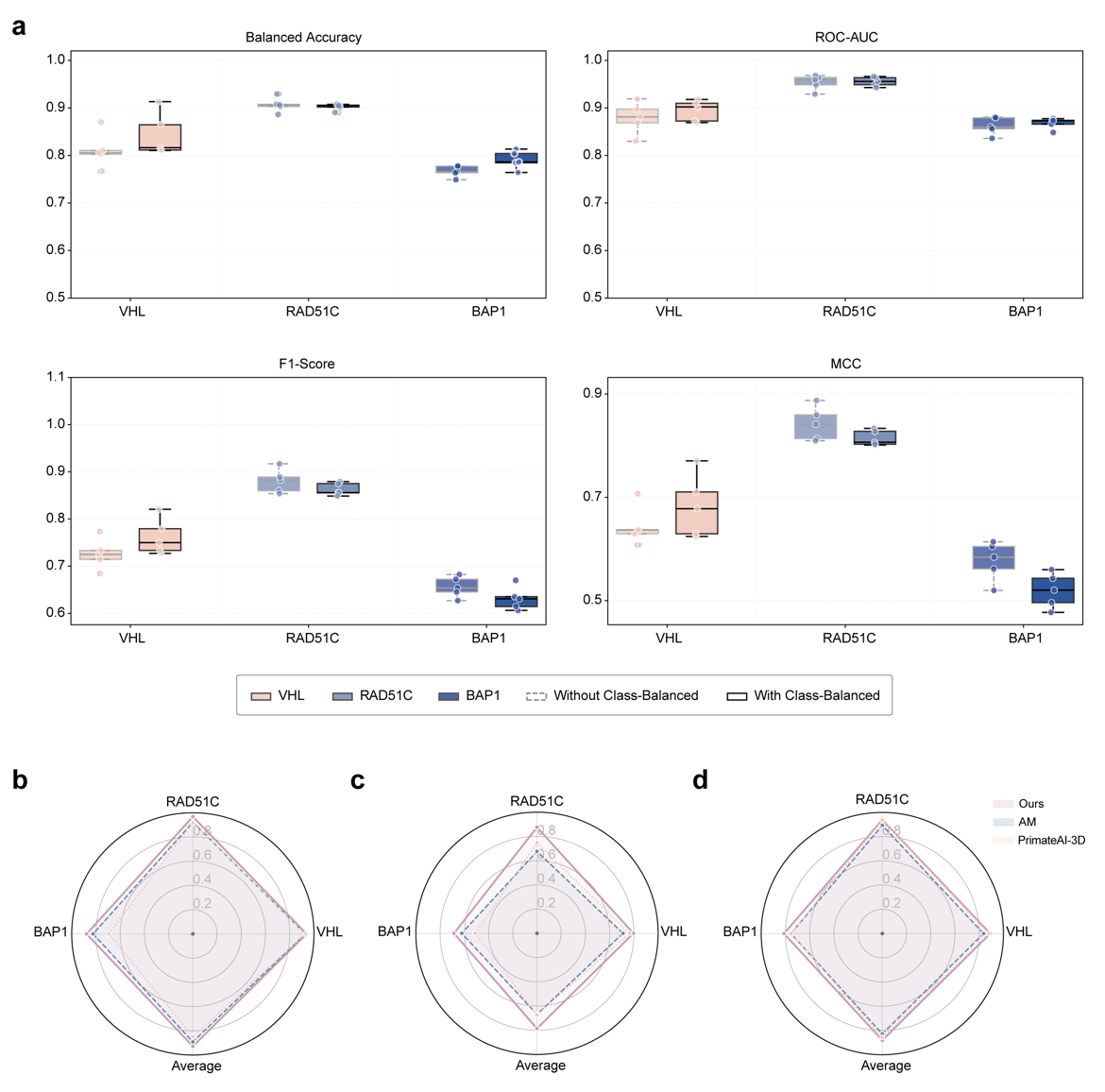


**Fig. S7** | **Performance evaluation of the proposed model across five other genes**.

**a,** Comparison of model performance with and without class balancing across five genes (VHL, ATM, BRCA1, RAD51C, BAP1), evaluated by Balanced Accuracy, ROC-AUC, F1-Score, and MCC. Box plots show results without (dashed) and with (solid) class balancing. **b-d**, Performance of our model across five disease-associated genes (BRCA1, ATM, VHL, BAP1, RAD51C) and their average, measured by ROC-AUC (**b**), F1-Score (**c**), and Accuracy (**d**). Radar plots illustrate the model's performance for genes with diverse mutation positions. Sample sizes for each gene are provided in Table S3.


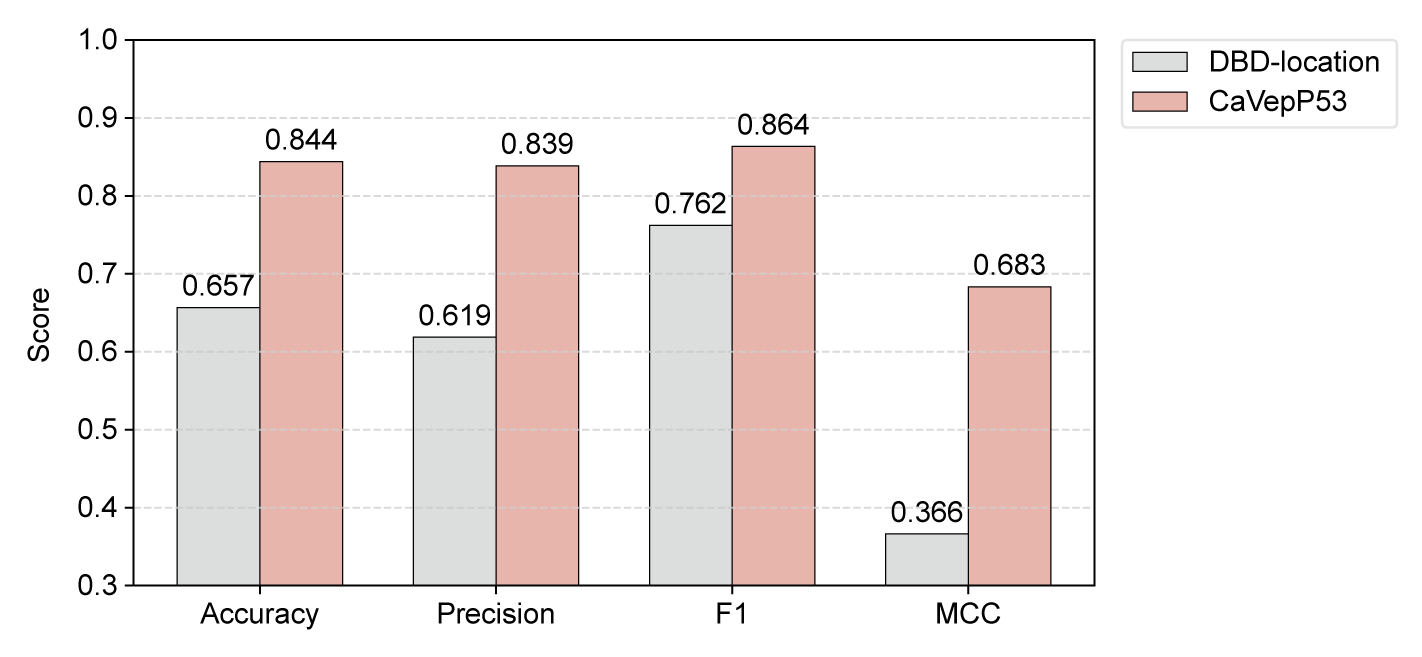


**Fig. S8 | Performance comparison of the DBD-location baseline and CaVepP53 across multiple metrics**. Bar plot showing Accuracy, Precision, F1‑score, and Matthews correlation coefficient (MCC) for the simple rule‑based classifier (DBD‑location, residues 102–292) and CaVepP53 model on the same test set (n = 503 variants).

**Table S1** | Statistical summary of the data sets

| Data set | The number of SAPs | | The number of SNPs | |
| --- | --- | --- | --- | --- |
|  | Benign | Pathogenic | Benign | Pathogenic |
| ClinVar set^*^ | 124 | 203 | 924 | 379 |
| NG set | 1464 | 1955 | 1189 | 730 |
| Total | 1546 | 1976 | 1836 | 827 |

*The ClinVar TP53 data was last updated on April 2, 2025. In the Single Amino Acid Polymorphisms (SAPs) data, ClinVar contains 3 duplicate entries, and the DMS dataset has 131 duplicates with 4 discrepancies in labeling (G187D, S269R, C277S, E285D). A total of 7 conflicting entries and 217 duplicate entries were found across both datasets, which were merged, deduplicated, and resolved for discrepancies. In the Single Nucleotide Polymorphisms (SNPs) data, ClinVar has no duplicates, while the DMS dataset contains 1 duplicate and 15 conflicting entries across both datasets, alongside 544 duplicates. These datasets were similarly merged, deduplicated, and discrepancies were addressed.

**Table S2** | **Performance comparison of models on the independent cBioPortal dataset (N = 503 TP53 missense variants).**

| Model | N | Mean score ± SD | | Cohen's d | ROC  ‑AUC |
| --- | --- | --- | --- | --- | --- |
|  | (driver/  non-driver) | driver | non-driver |  |  |
| CaVepP53 | 253 / 250 | 0.701 ± 0.371 | 0.090 ± 0.208 | **2.03** | **0.918** |
| Alphamissense | 253 / 250 | 0.736 ± 0.321 | 0.197 ± 0.227 | 1.94 | 0.893 |
| PrimateAI-3D | 233 / 246^*^ | 0.691 ± 0.176 | 0.425 ± 0.148 | 1.64 | 0.865 |

*PrimateAI-3D predictions were available for 479 variants (233 driver, 246 non‑driver); the remaining 24 variants lacked a value in the original data.

**Table S3** | **Statistical summary of datasets used for scalability validation across five additional genes.**

| Gene | DMS Dataset | | Clinvar Dataset | | Total | |
| --- | --- | --- | --- | --- | --- | --- |
|  | Benign | Pathogenic | Benign | Pathogenic | Benign | Pathogenic |
| ATM | 13195 | 3256 | 109 | 83 | 13304 | 3339 |
| BRCA1 | 470 | 1196 | 364 | 208 | 834 | 1404 |
| VHL | 740 | 245 | 33 | 159 | 748 | 261 |
| BAP1 | 3389 | 968 | 28 | 27 | 995 | 3417 |
| RAD51C | 1687 | 621 | 72 | 16 | 1759 | 637 |

**Table S4** | 22 TP53 mutants validated by experiments

| **Mutant** | **Type/source (Clinvar /Ensembl)** | **DMSO (%)** | **Nut3a (%)** |
| --- | --- | --- | --- |
| R110A |  | 42 | 67 |
| (Biological replicate) | | 36 | 74 |
| S116P | VUS (Ensembl) | 4 | 12 |
|  |  | 4 | 16 |
| C124S | VUS (Ensembl, ClinVar) | 8 | 9 |
|  |  | 11 | 10 |
| V218I |  | 22 | 57 |
|  |  | 30 | 53 |
| E224F |  | 30 | 72 |
|  |  | 27 | 66 |
| P250A | VUS (Ensembl, ClinVar) | 53 | 82 |
|  |  | 47 | 80 |
| L265I |  | 8 | 19 |
|  |  | 12 | 19 |
| T329H |  | 32 | 32 |
|  |  | 35 | 24 |
| Q52F |  | 32 | 41 |
|  |  | 29 | 48 |
| S90W |  | 10 | 17 |
|  |  | 10 | 15 |
| W91T |  | 7 | 15 |
|  |  | 7 | 19 |
| I254V | Likely benign (Ensembl, ClinVar) | 16 | 44 |
|  |  | 14 | 46 |
| E343L |  | 18 | 19 |
|  |  | 24 | 14 |
| K351A |  | 37 | 53 |
|  |  | 38 | 52 |
| S367P |  | 4 | 6 |
|  |  | 5 | 6 |
| S99Y | VUS (Ensembl) | 21 | 58 |
|  |  | 21 | 53 |
| T125R | Pathogenic (Ensembl, ClinVar) | 35 | 84 |
|  |  | 43 | 82 |
| G262D | VUS (Ensembl, ClinVar) | 61 | 98 |
|  |  | 63 | 99 |
| G334L |  | 33 | 66 |
|  |  | 38 | 66 |
| L348P |  | 41 | 88 |
|  |  | 40 | 84 |
| D61P |  | 47 | 51 |
|  |  | 52 | 58 |
| T312S | Benign (Ensembl, ClinVar) | 9 | 16 |
|  |  | 8 | 18 |

1. The rows highlighted in green represent positive reference data used in this experiment.

**Table S5** | The statistics of fine-tuned PLM prediction accuracy.

| Mutations | DMS Label | AM Label | Validation Labels | ESMC Score(pretrained) | ESM label (pretrained) | CaVepP53 Label | Confidence Score |
| --- | --- | --- | --- | --- | --- | --- | --- |
| Q52F | 2 | 0 | 0 | -2.5 | 0 | 0 | 0.996713996 |
| D61P | 2 | 0 | 0 | 0.875 | 0 | 0 | 0.999270141 |
| S90W | 2 | 0 | 1 | -3.859375 | 0 | 0 | 0.929608405 |
| W91T | 2 | 1 | 1 | 3.984375 | 0 | 0 | 0.999879837 |
| S99Y | 2 | 1 | 1 | -5.625 | 1 | 1 | 0.999272525 |
| R110A | 2 | 0 | 1 | -6.625 | 1 | 1 | 0.923024476 |
| S116P | 2 | 1 | 1 | -2.625 | 0 | 1 | 0.775534749 |
| C124S | 2 | 2 | 0 | -3.625 | 0 | 1 | 0.978271067 |
| T125R | 1 | 1 | 1 | -9.3125 | 1 | 1 | 0.987766743 |
| V218I | 0 | 0 | 1 | -3.984375 | 0 | 1 | 0.575175881 |
| E224F | 1 | 1 | 1 | -3.75 | 0 | 1 | 0.98059696 |
| P250A | 2 | 0 | 1 | -5.25 | 0 | 1 | 0.747959018 |
| I254V | 0 | 0 | 1 | -1.125 | 0 | 0 | 0.701236784 |
| G262D | 1 | 1 | 1 | -5.125 | 0 | 1 | 0.999925137 |
| L265I | 0 | 0 | 1 | -4 | 0 | 1 | 0.653255582 |
| T312S | 0 | 0 | 1 | 0.8671875 | 0 | 0 | 0.998555005 |
| T329H | 2 | 0 | 0 | -5 | 0 | 0 | 0.883608699 |
| G334L | 2 | 1 | 1 | -11.4375 | 1 | 1 | 0.961427212 |
| E343L | 2 | 0 | 0 | -0.4375 | 0 | 0 | 0.909023583 |
| L348P | 2 | 1 | 1 | -8.5625 | 1 | 0 | 0.975818515 |
| K351A | 2 | 2 | 1 | -2.1875 | 0 | 0 | 0.999624729 |
| S367P | 2 | 0 | 0 | 0.8671875 | 0 | 0 | 0.999918938 |
| Accuracy |  | 59.1% |  |  | 50.0% | 68.2% |  |

1. The cells in color indicate consistency between the model-predicted label and the experimentally validated label (Validation Labels)
